# Lumbar Muscle Fatty Infiltration and Atrophy in Patients with Low Back Pain and Degenerative Spinal Pathologies: A CT Imaging Study

**DOI:** 10.3390/jcm14062125

**Published:** 2025-03-20

**Authors:** Tess Mardulyn, Arnaud Delafontaine, Patrice Jissendi, Laurent Fabeck

**Affiliations:** 1 Orthopaedic Surgery Department, University Hospital Center Saint-Pierre, 1000 Bruxelles, Belgium; tessmardulyn@hotmail.com (T.M.); laurent.fabeck@ulb.be (L.F.); 2 Laboratory of Functional Anatomy, Faculty of Motor Sciences, Free University of Brussels (ULB), 1000 Brussels, Belgium; 3 Laboratory of Anatomy, Biomechanics and Organogenesis, Faculty of Medicine, Free University of Brussels (ULB), 1000 Brussels, Belgium; 4 Complexité, Innovation, Activités Motrices et Sportives (CIAMS), Université Paris-Saclay, 91404 Orsay, France; 5 Radiologic Department, University Hospital Center Saint-Pierre, 1000 Bruxelles, Belgium; patrice.jissendi.tchofo@ulb.be

**Keywords:** spine, low back pain, fatty infiltration, lumbosacral

## Abstract

**Background/Objectives:** Low back pain (LBP) may be related to intramuscular fatty infiltration (FI), the topography of which has been the subject of only a few studies. Our goal is therefore to determine the importance and topography of FI at the lumbar level and evaluate its correlation with LBP. **Methods:** We conducted a retrospective study and compared 254 LBP patients who underwent a lumbosacral CT scan with a sample of 115 healthy subjects, all classified into three age groups (≤35, 36–55, and >55 years old). In CT scan images from L2 to S1, muscle density (Hounsfield unit values ranging from −29 to +150), reflecting intramuscular FI, was measured. LBP was further divided into five subgroups of pathologies. **Results:** There was a significant difference in muscle density between the small and large circles at the L4/L5 and L5/S1 levels in LBP patients, which was not observed in the healthy subjects. In both LBP patients and healthy subjects, a decreasing density gradient was observed from L2 to S1, with a significant difference in density across age groups. LBP patients exhibit lower muscle densities compared to healthy subjects. **Conclusions:** In LBP patients, fatty infiltration (FI) of the paraspinal muscles is most pronounced in the lower lumbar region and appears to be localized at the level of muscle insertion. This localized muscle deficit differs from the age-related process of FI and may contribute to the development of LBP and discopathies.

## 1. Introduction

Low back pain (LBP) is a major health issue and the leading cause of disability worldwide [1,2,3,4,5]. The definition of low back pain encompasses discomfort in the posterior region of the body, extending from the lower edge of the 12th ribs to the lower gluteal folds, with or without radiating pain in one or both legs, lasting for at least one day [6].

Approximately two-thirds of adults will experience LBP at some point, most commonly between the ages of 30 and 50 [3,7,8]. Of these, 70% will experience recurrence, which often leads to a chronic condition [2,3]. Despite the high prevalence of this health issue and extensive research in this area, our understanding of the pathogenesis and biological determinants of LBP remains insufficient [9], and preventive methods are still lacking.

Paraspinal lumbar muscles have been the subject of extensive research using imaging techniques. However, there is limited information regarding their role in the etiology of LBP [10,11,12]. Lumbar muscle degeneration is commonly associated with non-specific LBP [13] and is characterized macroscopically by a reduction in muscle mass (atrophy) and an increase in fatty infiltration (FI), although the exact nature of this association remains unclear [5,14,15,16]. Some studies showed that muscle atrophy and FI are related to LBP [5,9,11,14,15,17,18,19,20,21,22,23,24], while others found no link between LBP and FI [10,12]. One explanation for these conflicting results is the influence of age and physical activity on muscle composition, volume, and density [10,16,19,20].

While some studies have attempted to demonstrate that pain leads to muscular atrophy [17], it would be interesting to explore the opposite hypothesis: that muscle loss at the vertebral level, not explained by fatty degeneration, may predispose individuals to low back pain (LBP). Some patients exhibit muscle atrophy but do not yet experience LBP or may never develop it.

Although it has been suggested that muscle deficit may precede pain, it is important to note that there are cases of discopathy and pain without muscle wasting, indicating that muscle wasting may not necessarily be caused by disk disease. Furthermore, many patients with LBP do not exhibit muscular degeneration, suggesting that other factors may contribute to the development of pain.

But how is lumbar fatty infiltration (FI) defined? Fatty degeneration is a well-known phenomenon of normal aging [12,16,19,20,21,22]. Similarly to the lumbar region, fatty involution at the shoulder level increases physiologically with age, with a significant acceleration after 40 years [20], and is a predictor of poor prognosis in the healing of rotator cuff tears [5,13,20]. Age is therefore a determining factor for FI [12,20,22].

FI at the shoulder and lumbar levels may appear to be the same degenerative process related to age. However, we can observe on the CT scans of LBP patients that muscle atrophy in the lower lumbar region is localized to areas in contact with the vertebra, whereas the entire muscle is affected at the level of the rotator cuff.

It is therefore legitimate to ask whether the classic degeneration observed in shoulder and lumbar muscles is not, in fact, two distinct phenomena. Muscle atrophy linked to LBP may have a distinct topography, suggesting that current muscle-building treatments for LBP may be less effective. The multifidus muscle, for instance, plays a significant role in maintaining lumbar spine stability, and its atrophy has been associated with the development of LBP [25,26]. The interspinous muscles, although smaller, also contribute to spinal extension and stability, and their degeneration can compromise the overall integrity of the spinal column, potentially leading to pain.

The objectives of this study are (1) to determine the importance and topography of FI at each of the last four lumbar levels by measuring muscle density in LBP patients and healthy subjects and (2) to evaluate the relationship between this lumbosacral FI and LBP, considering the possible effects of age, gender, intervertebral level, side, and the context of degenerative spinal conditions.

## 2. Materials and Methods

### 2.1. Methodology of Scientific Research

This is a retrospective study approved by the local ethics committee. All procedures involving human participants were conducted in accordance with the ethical standards of the institutional research committee (Reference Number: O.M. 007; Free University of Brussels) and with the 1996 ICH Guidelines.

#### 2.1.1. Selection of Patients

**Figure 1:**
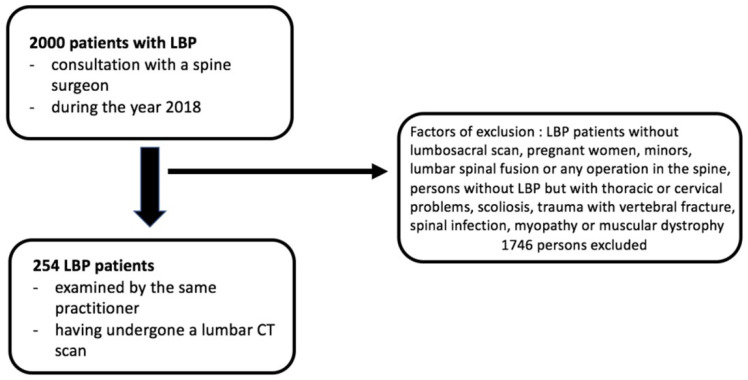
Diagram showing inclusion and exclusion criteria for LBP patients.

From 10 January 2023 to 30 September 2024, hundreds of patients with LBP attended orthopedic consultations. The medical records of each of these patients were reviewed. Only those who had undergone a lumbosacral scan and had suffered from LBP for more than 6 months were included in the study. The following were excluded: pregnant women, minors, patients with lumbar spinal fusion or any history of spinal surgery, individuals without LBP but with thoracic or cervical issues, those with severe structural deformities (e.g., scoliosis), victims of trauma with a recent vertebral fracture, patients with spinal infections (spondylodiscitis, osteomyelitis, or sacroiliitis), and those with myopathy or muscular dystrophy (Figure 1).

To develop comparisons with healthy control subjects, a large sample of non-lumbar patients who had undergone an abdominal or urological CT scan was also collected. The use of CT scans in the healthy control group was solely for the purpose of comparing lumbar muscle densities, and these patients were not selected based on any lumbar-related issues. These scans were performed for other clinical reasons (abdominal/urological conditions) that did not involve any lumbar pathology. Indeed, the indication for the scan was related to abdominal or urological pathologies. The medical records of each patient were then reviewed to ensure that they had never experienced low back pain. Additionally, each patient was contacted by phone to further confirm the absence of low back pain, ensuring their eligibility for inclusion as healthy subjects.

**Figure 2:**
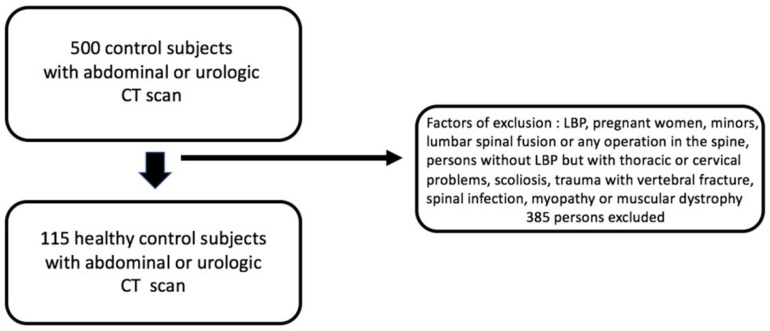
Diagram showing inclusion and exclusion criteria for healthy control subjects.

A list of patients who had undergone this type of scan between 10 January 2023 and 30 September 2024 was consulted. We randomly selected odd-numbered months, and a phone call was made to 500 patients to inquire whether they had or had ever had low back pain. The same exclusion criteria that were applied to LBP patients were also applied to the healthy subjects (Figure 2).

The subjects were classified into three age groups: young (≤35 years old), middle-aged (36–55 years), and elderly (>55 years). LBP subjects were further divided into five subgroups based on pathology: disk herniation, discopathy, narrow canal, spondylolysis/spondylolisthesis, and simple LBP (no identified pathology).

#### 2.1.2. Statistical Analysis

Statistical analysis was performed using R Statistical Software (v4.0.0; R Core Team, 2021). The statistical objective was to characterize muscle density by age, gender, and pathology in LBP patients, who were then compared to healthy subjects. For each subgroup, the density of the two types of circles (large and small), sides, and lumbar levels was compared.

Descriptive statistics: For each objective, we calculated the means and standard deviations for each parameter in the different subject groups, which were reported in the form of tables or histograms.

Inferential statistics: The normality of the data distribution was tested using the Kolmogorov–Smirnov test. The Levene test was then applied to assess the equality of variances. Since these conditions were met, an analysis of variance (repeated measures ANOVA) was conducted for each comparison. The Newman–Keuls post hoc test was used to evaluate pairwise interactions. Independent samples *t*-tests were also performed to compare the mean age between healthy subjects and subjects with low back pain for each age category to eliminate the potential effect of age on the results. A threshold value of *p* < 0.05 was used to determine statistical significance.

To test the reliability between the values of volume sections and reconstructed sections for LBP patients, Pearson correlation coefficients were calculated for each level, each side, and each circle. A Bland–Altman analysis was then performed by calculating the average differences and the 95% limits of agreement. To evaluate cross-examiner reproducibility, intraclass correlation coefficients were used.

### 2.2. CT Imaging Research Methodology

CT images were used to measure the densities, in Hounsfield units, of the paravertebral lumbar muscles. The average density reflects the degree of intramuscular fatty infiltration (FI), as density values decrease with increasing fat content [16,27,28].

All CT images were obtained using a multidetector computed tomography (MDCT) Siemens Somatom Definition AS scanner with 64 and 128 detectors (2017, Siemens, Erlangen, Germany). The software used for the analysis was Impax 6.5.2.114, provided by AGFA (AGFA Health Care N.V. 2011).

**Figure 3:**
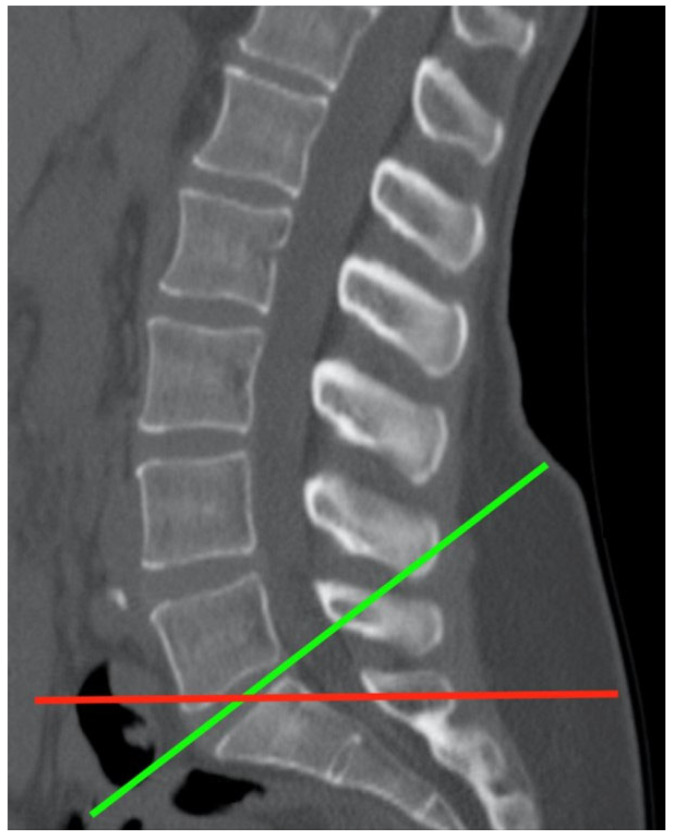
A sagittal cross-section of a lumbar spine CT scan: the red line indicates the volume slice and the green indicates the reconstructed slice.

Two types of sections can be performed on a lumbar spine CT scan: reconstructed and volume slices. Reconstructed slices follow the plane of each intervertebral disk and are usually used to evaluate spinal pathologies, while volume slices, typically used in other types of scans, represent conventional axial slices perpendicular to the patient’s axis (Figure 3). To ensure better correlation with the volume sections of abdominal and urological scans, both types of sections were examined on the lumbar spine CT scans.

We compared the native slice thickness images (2 mm) of the abdominal scans with the reformatted slice thickness images (2 mm/2 mm) of the lumbar scans. The analyses were performed using an MPR tool integrated into our PACS software (Impax 6.0). All image analyses were conducted by the same examiner, assisted by the head of the Imaging Department.

Measurements were made at four lumbar levels, from L2 to S1, at the vertebral body level, aligning with standard practices in musculoskeletal imaging. It is important to note that the measurement of muscle areas and densities using CT scans is typically performed at the vertebral body level. This approach is consistent with established methodologies in the literature [29]. For instance, a study demonstrated that the skeletal muscle index and psoas muscle index, measured at the third lumbar spine (L3) level, can predict osteoporosis [30].

**Figure 4:**
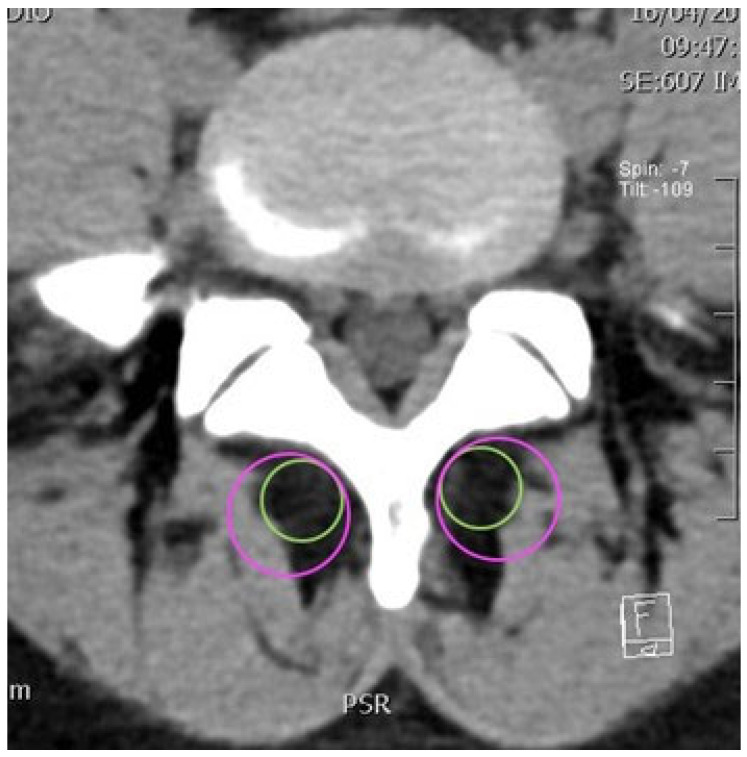
An axial cross-section of a lumbar CT scan with an illustration of the measurements of the large and small circles in pink and green, respectively.

The software allows for the use of a circle to define a studied area in which it can calculate the average density. A 2 cm^2^ circular area was used for the small circle, and a 5 cm^2^ area was used for the large circle (Figure 4). It is acknowledged that variations in body size could influence the measured muscle areas, particularly given that females tend to have smaller muscle sizes. Although we used standard 2 cm^2^ and 5 cm^2^ circles to assess muscle density, future studies may consider adjusting circle sizes based on individual body size to improve measurement accuracy.

On the left and right sides of the spine, the density of two standard muscle areas on CT scan slices was measured using two circumscribed circles, one large and one small. On each slice, the two circles were positioned as tangentially as possible, on the one hand, to the base of the spinous process, and on the other hand, to the posterior lamina, avoiding interference from bone density (Figure 4). The purpose of using circles of different sizes was to cover a small and a large portion of the muscles and obtain at least two different mean density values depending on the area covered. The two circles each encompass the muscle’s insertion on the vertebra and extend towards the muscle tissue. These circles of different sizes are useful for analyzing the topography of fatty infiltration, which may not be homogeneous and may be localized at the level of muscle insertion. Indeed, the hypothesis that the small circle is less dense than the large one would confirm that atrophy is localized at the level of muscle insertion.

To evaluate the reliability of the measurements taken by two examiners, a comparative sample containing the measurements from 5 randomly selected patients in the cohort—totaling 160 measurements—was carried out by two independent observers.

## 3. Results

The study was conducted with a total of 369 patients. A total of 254 LBP patients, examined by the same practitioner and having undergone a lumbar CT scan, were included (Figure 1). LBP was assessed using a Visual Analog Scale (VAS), and the average VAS score at the first consultation was 4.40. A total of 115 non-lumbar patients who had undergone abdominal or urologic CT scans were also included (Figure 2). The demographic characteristics of the LBP group are shown in Table 1. For healthy subjects aged ≤35 years (*n* = 31; 17 women and 14 men), the mean age was 27.6 ± 5.4 years. For healthy subjects aged between 36 and 55 years (*n* = 50; 16 women and 34 men), the mean age was 44.9 ± 5.9 years. For healthy subjects aged >55 years (*n* = 34; 11 women and 23 men), the mean age was 69.5 ± 9.1 years. Independent samples *t*-tests were conducted to compare the mean age of healthy subjects (27.6 ± 5.4 years) and subjects with low back pain (28.83 ± 6.04 years) in the ≤35-year-old age category (t(52) = −0.77; *p* > 0.05); the mean age of healthy subjects (44.9 ± 5.9 years) and subjects with low back pain (45.9 ± 5.22 years) in the 36–55 years age category (t(163) = −1.03; *p* > 0.05); and the mean age of healthy subjects (69.5 ± 9.1 years) and subjects with low back pain (66.28 ± 8.45 years) in the >55 years age category (t(148) = 1.84; *p* > 0.05), showing no significant differences.

Comparisons within the LBP group were made based on measurements from the reconstructed sections. Volume sections were used for comparisons within the control group and between the control group and the LBP group.

### 3.1. Low Back Pain Patients

**Figure 5:**
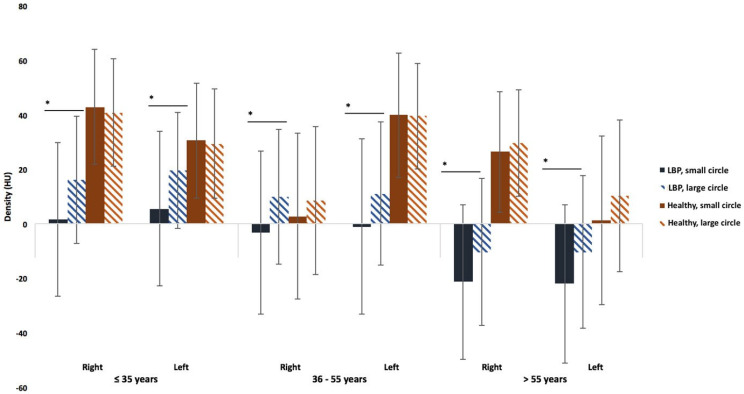
A comparison between the small circle and the large circle for LBP patients and healthy subjects for the 3 age categories (35 years, 36–55 years, and >55 years) at L5/S1. The “*” symbol indicates a significant difference between the circles.

For LBP patients, there is a statistically significant difference in muscle density between the small and large circles in the lower lumbar region (L4/L5, L5/S1) (*p* < 0.0463) (Figure 5).

**Figure 6:**
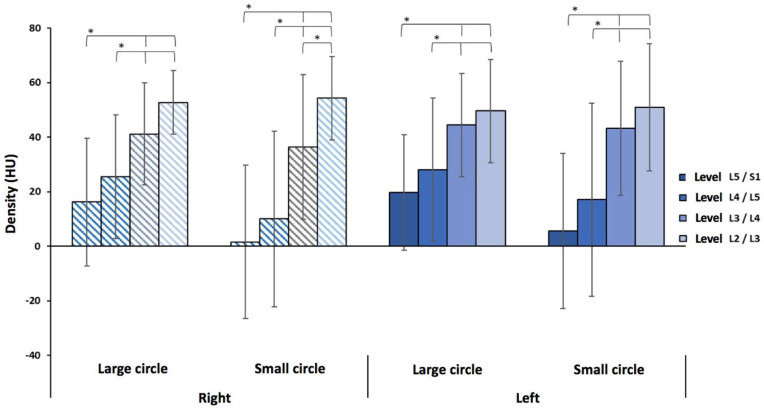
A comparison between levels for low back pain patients for the ≤ 35 years age group. The “*” symbol indicates a significant difference in density (*p* < 0.05) between two levels; the braces indicate the differences from L5/S1 and L4/L5 compared to the other levels.

When comparing levels, there is a decreasing density gradient from L2 to S1, with lower muscle densities at the last two lumbar levels for both large and small circles (Figure 6). Indeed, in the three age groups, there is a statistically significant difference in density at L5/S1 and L4/L5 compared to the other levels (*p* < 0.0001).

**Figure 7:**
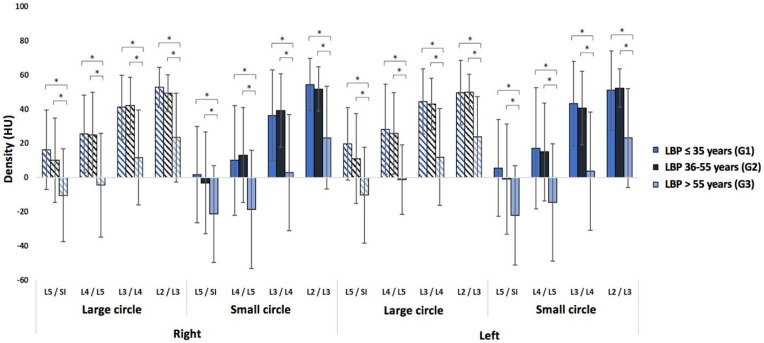
A comparison of three age groups (≤35, 36–55, and >55 years) for low back pain patients. The “*” symbol indicates a significant difference between two groups.

Regardless of the circle size or lumbar level, there is a significant difference in muscle density between the younger and older age groups and between the middle-aged and older age groups (*p* < 0.0001) (Figure 7). We also observe a significant difference (*p* < 0.0001) between men and women for all parameters. However, there is no difference in density between the right and left sides of the spine.

Regardless of the pathology, there is no difference in muscle density in LBP patients, except for those with a narrow canal (Table 2).

### 3.2. Healthy Subjects

In the healthy subjects, a decreasing density gradient from L2 to S1 is also observed for both circles. There is a statistically significant difference in density between L5/S1 and L2/L3 (*p* < 0.0190) and between L5/S1 and L3/L4 (*p* < 0.0249). However, regardless of the lumbar level, no difference in density is observed between the small and large circles (Figure 5).

**Figure 8:**
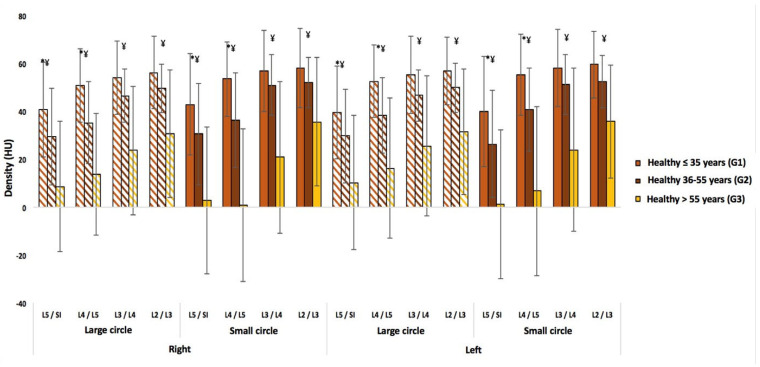
A comparison of three age groups (≤35, 36–55, and >55 years) for healthy subjects. The “*” symbol indicates a significant difference between the young and middle-aged groups, while the “¥” symbol indicates a significant difference between the young and older age groups and between the middle and older age groups.

Regardless of the circle size or lumbar level, a statistically significant difference in density is found between each age group (*p* < 0.0001), except in the upper lumbar region (L2/L3 and L3/L4) between the younger and middle-aged groups (Figure 8). On the other hand, no asymmetry between sides or differences between sexes is observed in the healthy subjects.

### 3.3. Comparisons Between the Healthy Subjects and the LBP Patients

**Figure 9:**
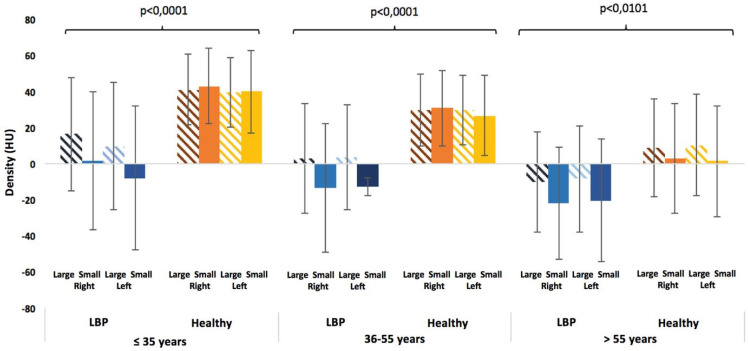
A comparison between low back pain patients and healthy subjects at the L5/S1 level for the 3 age categories (≤35 vs. 36–55 vs. >55 years). The braces indicate a significant difference in the various conditions (sides and circles) between the groups.

At each lumbar level, muscle densities of large and small circles were compared on the volume slices for both LBP patients and healthy subjects. In LBP patients, a statistically significant difference in density between the small and large circles was observed in the lower lumbar region (L4/L5 and L5/S1) (*p* < 0.0463), with the small circle showing lower density values than the large one (Figure 5 and Figure 9). In contrast, at all lumbar levels in healthy subjects, the densities of the large and small circles were not significantly different (Figure 5 and Figure 9).

Regardless of the circle size or lumbar level, a statistically significant difference in muscle density was observed between LBP patients and healthy subjects for all age groups (*p* < 0.0468), with LBP patients having lower muscle densities than healthy subjects, except for the middle-aged group in the upper lumbar region (L2/L3 and L3/L4) (Figure 9).

### 3.4. Reliability of Measurements

The results demonstrate excellent cross-examiner reproducibility, with ICC values ranging from 0.84 to 0.99 for the different comparisons.

Regarding the comparison of the lumbosacral scan values, all correlations were significant, with r = 0.95 for the strongest correlation and r = 0.49 for the weakest correlation. Furthermore, the systematic error was minimal, further confirming the reliability of the parameters.

## 4. Discussion

The high prevalence of LBP has prompted the scientific community to investigate the origins of this health issue. The primary aim of this study was to determine the topography of fatty infiltration (FI) at the lumbar level and to demonstrate that the lumbar atrophy observed is distinct from physiological age-related degeneration.

**Figure 10:**
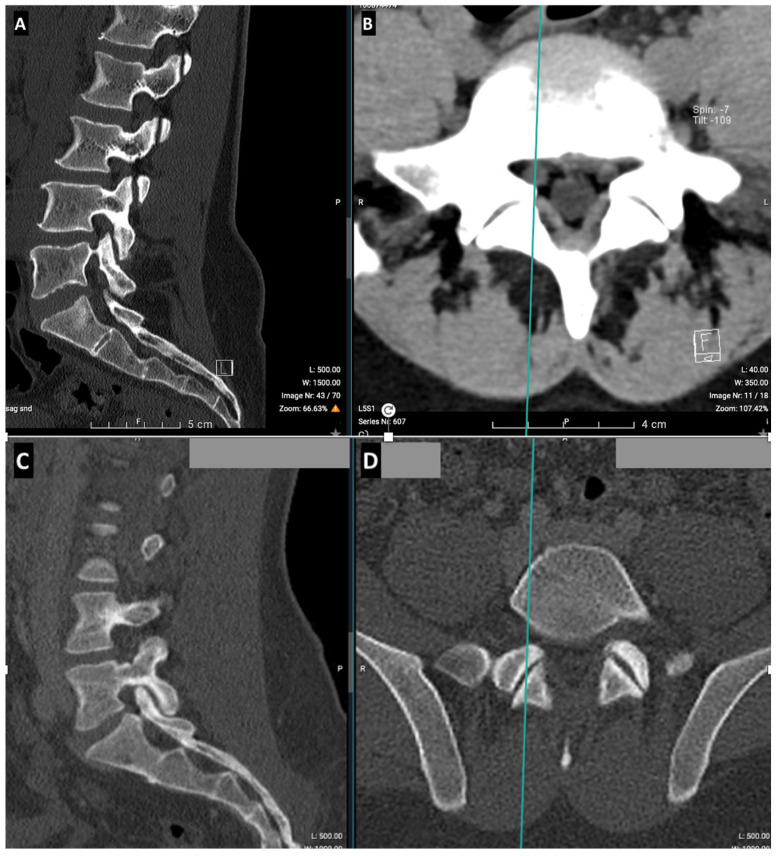
Muscle deficit localized at the level of the insertion of the muscle in the low lumbar region (L5/S1), seen in the sagittal section (**A**) and in the axial section (**B**) in a low back pain patient, compared to a non-deficient muscle in a healthy subject, seen in the sagittal section (**C**) and in the axial section (**D**).

Interestingly, our study reveals that in LBP patients, there is a significant difference in density between the muscle insertion area on the vertebra (small circle) and the muscle belly (large circle) in the lower lumbar region (L4/L5 and L5/S1), while no statistically significant difference in density is observed between these two areas in the upper lumbar region (L2/L3 and L3/L4). Since the small circle reflects lower density values than the large one—indicating reduced density at the bone contact—our hypothesis that atrophy is localized at the level of muscle insertion appears to be confirmed (Figure 10). The FI in the extensor muscles of the spine manifests in a non-uniform pattern, distinguishing it from normal physiological muscle degeneration. This phenomenon is not observed in healthy subjects in whom the muscle density is similar at both the belly and insertion.

From a broader topographical perspective, our findings align with those of references [12,14,15,16,17,19,28,29,30,31], which show that muscles appear more atrophied at the last two lumbar levels.

Our results also corroborate much of the existing literature [5,14,15,17,18,24,30], demonstrating that fatty infiltration (FI) is significantly higher in the paraspinal muscles of LBP patients compared to healthy subjects. Some authors have thus hypothesized that LBP and pain-related inactivity contribute to atrophy [17,30]. However, the muscular deficit may precede the onset of pain. While some studies [15,16] found atrophy in the extensor muscles of the lumbar spine, they observed no atrophy in the hip and spinal flexors, suggesting that the atrophy seen in the extensors was not merely a result of general inactivity. This supports our hypothesis that muscle atrophy exhibits a localized topography (Figure 10). However, it is important to note that the multifidus muscle increases in size from L2 to S1, which may influence the observed decrease in muscle density. While our study focused on muscle density, further research could benefit from normalizing muscle density values based on a cross-sectional area to account for these anatomical variations. It should be noted that a CT scan does not allow for the isolation of each muscle group unlike MRI. Based on a CT scan alone, it is difficult to analyze the boundary of the multifidus muscle. Therefore, density measurements were performed for the entire lumbar muscle group.

The loss of lumbar muscles at the level of their insertion on the vertebra supports the hypothesis that muscle deficit could predispose individuals to LBP. Indeed, if LBP was the cause of atrophy, it would likely manifest in a uniform manner, affecting all muscles (as seen in the rotator cuff) [20].

Very few studies have attempted to demonstrate that muscle atrophy causes LBP rather than the other way around. Danneels et al. [15] were among the first to suggest that muscular atrophy could play an etiological role in the development of LBP and potentially precede the onset of pain. Two studies, prior to ours, sought to understand the role of lumbar muscle FI as a potential biological factor responsible for the future development of LBP. Hebert et al. [18] hypothesized that a larger FI in the multifidus muscle would be associated with a higher likelihood of developing LBP. They found a positive association between FI and LBP but were unable to demonstrate that FI could be a predictor of future LBP. A possible explanation for this finding is the positive relationship between the age and FI of skeletal muscle, suggesting that FI may appear earlier in people with LBP but later become a common occurrence in older adults. Therefore, if there is a clinically relevant relationship between FI and LBP development, and since fat accumulation in muscles is a natural consequence of aging, age could confound the relationship between FI and LBP [19]. Fortin et al. [24] demonstrated that lower L5/S1 fatty tissue was associated with a decreased likelihood of suffering from LBP. These results suggest that a decrease in lumbar muscle quality, as assessed by the presence of FI, is linked to a higher risk of experiencing continuous, frequent, and persistent LBP after one year of follow-up.

Moreover, according to the results of our study, the role of pathology in assessing lumbar FI appears to be of limited relevance. Indeed, regardless of the pathology in LBP patients, no significant difference in muscle density was observed, except for those with a narrow canal. This is understandable, as a narrow lumbar canal primarily affects the elderly. Age therefore plays a more significant role in FI, and the observed difference in density for this pathology could be attributed to the greater impact of physiological fatty degeneration.

Our study shows that age influences muscle density. Whether in LBP patients or healthy subjects, FI increases with age and is particularly noticeable in those aged >55 years. This finding confirms, in agreement with several studies [12,17,19,22,31,32,33], that fatty degeneration at the lumbar level is a normal aging phenomenon. However, for all age groups included in our study (including the very young), LBP patients have significantly lower muscle densities than healthy subjects. This suggests that there is another factor, in addition to the physiological process of aging, that contributes to the occurrence of LBP.

Our results also show that women with LBP have lower muscle density than men. This finding has been reported in several studies [10,17,18,19]. However, we do not observe any difference between the sexes in healthy subjects. This challenges the often-cited explanation by various authors that the gender disparity may be related to differences in body composition, with women having a higher proportion of body fat [17]. In this study, we observed significant differences in muscle density and fatty infiltration of the lumbar paraspinal muscles based on age and sex. The results indicate a general trend toward greater fatty infiltration in females compared to males, as well as variations in muscle density across different lumbar levels. However, it is important to note that these observations should be interpreted with caution due to certain methodological limitations, particularly the lack of multivariate analysis to independently assess the impact of age, sex, and other confounding factors.

Our findings show a clear relationship between age and fatty infiltration of the lumbar paraspinal muscles, with a tendency for lower muscle density in the older age groups, both in low back pain (LBP) patients and healthy controls. This is in line with the existing literature that suggests that aging leads to progressive muscle mass loss and increased fatty infiltration, a phenomenon known as sarcopenia. However, the impact of aging on the progression of fatty infiltration appears to be more pronounced in LBP patients, which could indicate a complex interaction between aging and the pathophysiology of low back pain.

Regarding sex differences, our results suggest that females have greater fatty infiltration than males. This could be attributed to sex-specific physiological factors, such as body composition, with females typically having a higher body fat percentage. Additionally, hormonal changes related to menopause may play a crucial role in the increase in fatty infiltration and muscle loss, particularly in postmenopausal sarcopenia, which warrants further investigation in future studies. We acknowledge that the analysis of these factors could be further enhanced by a multivariate approach, considering the independent effects of age, sex, body fat percentage, and other potential physiological factors. Such an approach would help better isolate the impact of each variable on fatty infiltration and muscle density of the lumbar paraspinal muscles. It would also clarify whether the observed sex differences are primarily related to hormonal factors or whether other underlying mechanisms related to body composition are at play.

Since MRI is considered the gold standard for soft tissue analysis, several authors [6,17] often use this modality to qualitatively assess fatty infiltration (FI) at the lumbar level using visual scales. While they have demonstrated a strong correlation between low back pain (LBP) and low back muscle FI, these authors recommend employing quantitative measures to assess the degree of FI [17,28]. In our study, we used muscle density as a variable. While both CT and MRI are valuable tools for assessing lumbar muscles, MRI is commonly used for visualizing fatty infiltration using well-established thresholding methods. However, CT was chosen for our study due to its ability to provide direct, quantitative measurements of muscle density in Hounsfield units, which allows for a more precise and objective assessment of fat content.

While physiotherapy is widely used for strengthening exercises in the rehabilitation of low back pain (LBP), it remains uncertain whether it can fully reverse muscle atrophy or specifically address localized muscle deficits associated with pain. Our findings suggest that muscle loss may not always be fully reversible through strengthening alone, underscoring the need for further investigation into the most effective treatment strategies for muscle recovery in LBP patients.

It is necessary to investigate whether a link exists between localized and focal muscle loss at the last two lumbar levels and the development of discopathies, particularly in the lower lumbar region. Further studies are needed to understand why these two phenomena are preferentially located at the last two lumbar levels.

This study has some weaknesses. Its retrospective nature limits the level of evidence. Additionally, there is a potential bias in the recruitment of healthy subjects, as they were gathered through telephone calls, which carries the risk of misunderstandings on their part. Another limitation of this study is the absence of longitudinal or postoperative data, which prevents us from determining whether fatty infiltration is a cause or a consequence of LBP. Future longitudinal studies are needed to clarify the temporal relationship between fatty infiltration and LBP progression. 

Regarding other limitations of our methodology, the choice of specific areas (i.e., the selection of 2 cm^2^ and 5 cm^2^ areas for the small and large circles, respectively) was based on established methodologies in the field of computed tomography (CT) imaging and body composition analysis. In CT imaging, the assessment of skeletal muscle area (SMA) is commonly performed at the third lumbar vertebra (L3) level, where muscle measurements are taken within predefined regions of interest (ROIs). These ROIs are typically circular or oval in shape, with their size and placement determined based on anatomical landmarks and the specific objectives of the study. For instance, a study by Somasundaram et al. [34] utilized automated deep learning pipelines to define normal ranges of skeletal muscle area and skeletal muscle index in children on CT, highlighting the importance of standardized ROI measurements in such analyses. The selection of 2 cm^2^ and 5 cm^2^ areas for the small and large circles, respectively, aligns with common practices in body composition research. These sizes are chosen to encompass muscle regions that are representative of the overall muscle mass at the L3 level, facilitating accurate assessments of muscle quantity and quality. For example, a study by van der Werf et al. [35] examined the agreement between non-contrast and contrast CT scan measurements of skeletal muscle area and mean attenuation, underscoring the significance of consistent ROI sizing in CT-based muscle analyses. The circle size method employed in this study has certain limitations. While previous research has established methods for muscle imaging, the use of 2 cm^2^ and 5 cm^2^ circles specifically for density measurement in LBP patients has not been extensively validated. Future research should explore adjustments in circle sizes based on individual patient characteristics such as body size and muscle anatomy to improve measurement accuracy.

Concerning our methodological analysis, it would be more efficient and reliable to expand our comparative sample size, focus on a single vertebral level (L4–5), and incorporate assessments from two independent observers. These modifications could enhance the inter-observer reliability of our study.

Concerning our statistical analysis, we did not conduct a multivariate analysis to assess age- and sex-related differences in muscle density. Moreover, no statistical comparison was performed regarding fatty infiltration between LBP patients with and without discopathies. Future research should address this limitation using appropriate statistical models.

The strengths of our study include the large number of LBP subjects of all ages, ensuring optimal statistical power, and the ability to compare them to a control (healthy) group. All subjects were examined by the same practitioner, a spine surgeon, and all data in this study were derived from objective measurements taken from CT scan images obtained at the same hospital.

Recent research has highlighted the significance of lumbar paraspinal muscle health in relation to low back pain. Ekşi and Özcan-Ekşi [36] found that fatty infiltration of the erector spinae at the upper lumbar spine could serve as a landmark for low back pain. Additionally, Wesselink et al. [37] utilized an automated computer vision model to investigate associations between lumbar paraspinal muscle health and factors such as age, BMI, sex, physical activity, and back pain. These findings underscore the importance of assessing muscle composition and health in the context of low back pain. Our study contributes to this body of knowledge by providing insights into muscle density measurements using CT imaging, which can help in understanding the role of muscle health in low back pain.

This could also facilitate the development of personalized rehabilitation protocols, with the caveat that the reversibility of fatty infiltration in lumbar paraspinal muscles remains to be demonstrated [38].

Our approach provides valuable insights for the implementation of Enhanced Recovery After Surgery (ERAS) protocols, particularly in stratifying LBP patients for day surgery versus prolonged hospitalization. This aligns with the findings obtained by Hoppe et al. [39], who demonstrated the relevance of 3D fatty infiltration analysis for prehabilitation and postoperative rehabilitation strategies in surgical candidates. Furthermore, the work of Zaed et al. [40] highlights the current lack of homogeneous patient selection criteria for ERAS protocols in degenerative lumbar spine surgery, emphasizing the need for objective assessment tools such as those proposed in our study.

## 5. Conclusions

While our study suggests that fatty infiltration of the lumbar paraspinal muscles may exhibit localized patterns in low back pain, the interpretation of this finding is limited by methodological constraints, including the small number of control cases and the heterogeneous causes of low back pain. Further research with larger, more homogeneous cohorts is needed to confirm whether localized muscle loss is a consistent feature of low back pain across various patient populations.

Atrophy, localized at the level of muscle insertion, appears to be more pronounced in the lower lumbar region. It may then represent an isolated muscle deficit at the vertebral insertion, distinguishing itself from the age-related fatty degeneration process, and could play a key role in the development of low back pain and discopathies.

Future studies should investigate the specific impact of discopathies on muscle fatty infiltration and clarify whether fatty infiltration exacerbates the progression of spinal degeneration or results from it. Additionally, exploring the impact of muscle fatty infiltration on postoperative outcomes and recovery after spinal surgery is a crucial clinical question. Longitudinal research comparing preoperative and postoperative muscle fatty infiltration could provide valuable insights into its potential role in recovery and rehabilitation following spinal surgery.

## Figures and Tables

**Table 1:** Demographic characteristics of low back pain patients.

	LBP Patients by Age (*n* = 254)			LBP Patients by Gender (*n* = 254)	
	≤35 Years (*n* = 23)	36–55 Years (*n* = 115)	>55 Years (*n* = 116)	Men (*n* = 108)	Women (*n* = 146)
Age	28.8 ± 6.0	45.9 ± 5.2	66.3 ± 8.5	50.1 ± 13.0	56.3 ± 14.7
Disc Herniation	39.1%	22.8%	6.1%	22.4%	12.5%
Discopathy	13.0%	44.7%	29.8%	31.8%	37.5%
Simple LBP (no pathology)	21.7%	16.7%	7.9%	15.9%	11.1%
Spondylolysis	8.7%	4.4%	2.6%	4.7%	3.5%
Spondylolisthesis	8.7%	3.5%	7.0%	4.7%	6.3%
Narrow Canal	8.7%	7.9%	46.5%	20.6%	29.2%

**Table 2:** Pathology classes: descriptive statistical results between pathology for raw data (density measurement in Hounsfield units).

			Narrow Canal (*n* = 64)	Discopathy (*n* = 88)	Disc Herniation (*n* = 42)	Simple LBP (*n* = 33)	Spondylolysis/Listhesis
RIGHT	Large circle	**L5/SI**	−8.66 ± 29.23	2.22 ± 27.00	6.55 ± 26.03	9.02 ± 27.94	5.57 ± 25.57
		**L4/L5**	17.11 ± 33.79	14.20 ± 29.09	22.40 ± 21.93	20.91 ± 27.42	5.96 ± 27.54
		**L3/L4**	14.86 ± 31.99	30.87 ± 25.72	38.81 ± 16.11	48.70 ± 13.15	39.82 ± 27.18
		**L2/L3**	22.49 ± 29.20	39.52 ± 20.15	35.25 ± 16.57	48.75 ± 13.15	39.82 ± 27.18
	Small circle	**L5/SI**	−18.36 ± 30.10	2.58 ± 33.19	8.70 ± 34.21	9.30 ± 23.90	37.41 ± 32.59
		**L4/L5**	17.87 ± 37.87	20.64 ± 32.73	33.25 ± 16.75	35.29 ± 21.16	46.40 ± 43.63
		**L2/L3**	23.36 ± 32.01	3.15 ± 28.11	7.37 ± 30.20	9.16 ± 29.19	37.41 ± 32.59
LEFT	Large circle	**L5/SI**	−8.51 ± 29.16	3.15 ± 28.11	7.37 ± 30.20	9.16 ± 29.19	39.82 ± 27.18
		**L4/L5**	14.44 ± 32.87	31.47 ± 23.87	19.11 ± 18.92	9.18 ± 11.89	37.41 ± 32.59
	Small circle	**L5/SI**	−19.38 ± 36.10	5.11 ± 33.29	37.06 ± 34.15	37.06 ± 34.15	16.43 ± 33.90
		**L4/L5**	17.87 ± 39.61	26.71 ± 32.16	37.06 ± 34.15	47.54 ± 20.48	39.07 ± 25.99
		**L2/L3**	23.94 ± 32.74	41.33 ± 23.05	50.61 ± 12.78	47.54 ± 20.48	39.07 ± 25.99

## Data Availability

The original contributions presented in this study are included in the article. Further inquiries can be directed to the corresponding author.
